# Association between neighborhood disadvantage and fulfillment of desired postpartum sterilization

**DOI:** 10.1186/s12889-020-09540-5

**Published:** 2020-09-22

**Authors:** Kavita Shah Arora, Mustafa Ascha, Barbara Wilkinson, Emily Verbus, Mary Montague, Jane Morris, Douglas Einstadter

**Affiliations:** 1 Department of Obstetrics and Gynecology, MetroHealth Medical Center, Case Western Reserve University, Cleveland, OH USA; 2 Cleveland Institute for Computational Biology, Case Western Reserve University, Cleveland, OH USA; 3 School of Medicine, Case Western Reserve University, Cleveland, OH USA; 4 Center for Health Care Research and Policy and the Departments of Medicine, and Population and Quantitative Health Sciences, MetroHealth Medical Center, Case Western Reserve University, Cleveland, OH USA

**Keywords:** Sterilization, Neighborhood disadvantage, Social determinants of health, Medicaid, Postpartum contraception

## Abstract

**Background:**

Adequacy of prenatal care is associated with fulfillment of postpartum sterilization requests, though it is unclear whether this relationship is indicative of broader social and structural determinants of health or reflects the mandatory Medicaid waiting period required before sterilization can occur. We evaluated the relationship between neighborhood disadvantage (operationalized by the Area Deprivation Index; ADI) and the likelihood of undergoing postpartum sterilization.

**Methods:**

Secondary analysis of a single-center retrospective cohort study examining 8654 postpartum patients from 2012 to 2014, of whom 1332 (15.4%) desired postpartum sterilization (as abstracted from the medical record at time of delivery hospitalization discharge) and for whom ADI could be calculated via geocoding their home address. We determined the association between ADI and sterilization completion, postpartum visit attendance, and subsequent pregnancy within 365 days of delivery via logistic regression and time to sterilization via Cox proportional hazards regression.

**Results:**

Of the 1332 patients included in the analysis, patients living in more disadvantaged neighborhoods were more likely to be younger, more parous, delivered vaginally, Black, unmarried, not college educated, and insured via Medicaid. Compared to patients living in less disadvantaged areas, patients living in more disadvantaged areas were less likely to obtain sterilization (44.8% vs. 53.5%, OR 0.84, 95% CI 0.75–0.93), experienced greater delays in the time to sterilization (HR 1.23, 95% CI 1.06–1.44), were less likely to attend postpartum care (58.9% vs 68.9%, OR 0.86, CI 0.79–0.93), and were more likely to have a subsequent pregnancy within a year of delivery (15.1% vs 10.4%, OR 1.56, 95% CI 1.10–1.94). In insurance-stratified analysis, for patients with Medicaid, but not private insurance, as neighborhood disadvantage increased, the rate of postpartum sterilization decreased. The rate of subsequent pregnancy was positively associated with neighborhood disadvantage for both Medicaid as well as privately insured patients.

**Conclusion:**

Living in an area with increased neighborhood disadvantage is associated with worse outcomes in terms of desired postpartum sterilization, especially for patients with Medicaid insurance. While revising the Medicaid sterilization policy is important, addressing social determinants of health may also play a powerful role in reducing inequities in fulfillment of postpartum sterilization.

## Background

Sterilization is the most commonly used method of contraception among women of reproductive age in the United States [1]. Patients with Medicaid insurance are less likely to undergo desired postpartum sterilization and more likely to have subsequent pregnancies than patients with private insurance [2–5]. This is, in part, due to a federal Medicaid policy that requires a specific consent form and a mandatory waiting period prior to sterilization due to a history of coerced sterilization of women of color and low socioeconomic status [4, 6–8]. However, barriers at the patient, physician, and hospital levels may also impact sterilization access and completion [4, 9]. In fact, after controlling for patient and physician level factors, we have found that the disparity in sterilization completion between patients with Medicaid and private insurance desiring postpartum sterilization is no longer significant [7]. Rather, Medicaid insurance status may be simply a proxy for other demographic and clinical factors such as adequacy of prenatal care and route of delivery that account for the difference in sterilization for patients with private versus Medicaid insurance [10].

Adequacy of prenatal care may reflect broader social determinants of health such as structural, financial, or psychosocial factors that limit access to the recommended number of prenatal visits such as distance to clinic, inconvenient hours, lack of transportation, childcare, distrust of physicians, and intimate partner violence, among others [11, 12]. The area deprivation index (ADI), first described by Singh, is a composite area-based index used to describe an area’s socioeconomic position and has been used to estimate the combined impact of individual and structural exposures that relate to the social determinants of health [13, 14]. The original ADI was constructed using factor analysis and principal-components analysis of 17 census indicators, including population aged ≥25 years with < 9 years of education, population aged ≥25 years with at least a high school diploma, employed persons aged ≥16 years in white collar occupations, median family income, income disparity, median home value, median gross rent, median monthly mortgage, owner occupied housing units, civilian labor force population aged ≥16 years unemployed, percentage of families below the poverty level, percentage of the population < 150% of the poverty threshold, percentage of single-parent households with children aged < 18 years, percentage of households without a motor vehicle, percentage of households without a telephone, percentage of occupied housing units without complete plumbing, and percentage of households with more than one person per room [13]. Factor score coefficients are used to weight the indicators comprising the index and the ADI is standardized by arbitrarily setting the mean at 100 and the standard deviation at 20. Higher levels of ADI (indicating greater area disadvantage), are independently associated with worse health outcomes in terms of acute and chronic health diseases, decreased healthcare participation, increased cost of care, and increased all-cause mortality [15–29]. Neighborhood disadvantage has been shown to impact health separately from individual health behaviors [24, 30]. Specifically in terms of pregnancy outcomes, neighborhood disadvantage is associated with cytomegalovirus seroprevalence, incidence of orofacial clefts, small and large for gestational age fetal growth, and preterm birth [31–38].

However, it is unclear whether neighborhood disadvantage impacts contraceptive access in general and more specifically, postpartum sterilization. The known association of sterilization fulfillment with adequacy of prenatal care may reflect the role of social determinants of health but also may simply reflect the mandatory waiting period prior to sterilization for patients with Medicaid insurance [39]. Thus, our objective was to analyze the relationship between neighborhood disadvantage and desired postpartum sterilization to better understand the impact of social determinants of health. We hypothesized that higher levels of neighborhood disadvantage (reflecting living in areas with fewer resources) as measured by the ADI, would be associated with decreased rates of sterilization achievement, increased time to sterilization, decreased postpartum visit attendance, and increased rates of subsequent pregnancy in the year following delivery. Furthermore, we hypothesized that this relationship would be observed within insurance-specific analyses as well, such that patients with Medicaid insurance would be more impacted by living in neighborhoods with fewer resources than those with private insurance, given the additional policy-level barriers to sterilization faced by patients with Medicaid.

## Methods

**Fig. 1:**
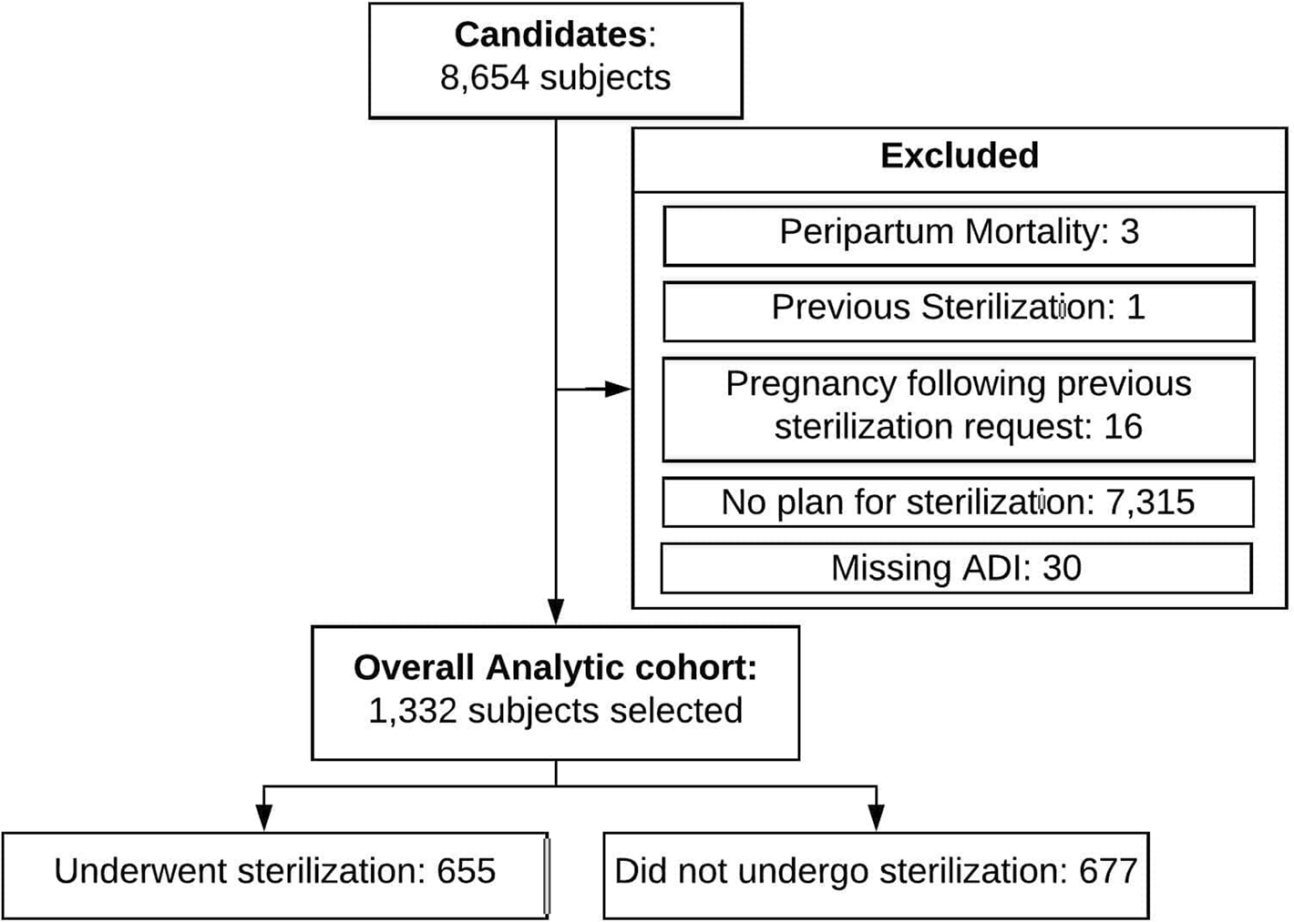
Flow chart of the study population

This is a planned secondary analysis of a retrospective cohort study including deliveries at or above 20 weeks gestation occurring from January 1, 2012 through December 31, 2014 at our tertiary care academic hospital. While the primary analysis restricted analysis of differences in postpartum sterilization fulfillment solely between patients with Medicaid versus private insurance, the cohort for this secondary analysis was broadened to include all patients whose postpartum contraceptive plan was sterilization and for whom ADI could be calculated (*n* = 1332) (Fig. 1). Sterilizations included those that occurred either during the delivery admission while inpatient or as an outpatient procedure during the first 90 days of the postpartum period.

Full study methodology has been published previously [10]. Briefly, we reviewed each patient’s linked outpatient and inpatient electronic medical record for demographic and clinical characteristics, postpartum contraceptive plan choice, and plan achievement for up to 365 days from date of delivery. Demographic and clinical characteristics recorded included maternal age, parity, gestational age, delivery type, number of prenatal visits, route of delivery, race and ethnicity, marital status, and education. Adequate prenatal care was defined as six or more prenatal visits [40]. Postpartum contraceptive plan was defined as the plan documented at time of hospital discharge. All study subjects had a contraceptive plan documented prior to discharge after delivery. Insurance status was directly obtained by matching patient charts to billing records.

We used ArcGIS (version 10.6.1) to geocode each subject’s home address at the time of delivery to obtain the census tract. Most (97%) of our subject addresses were matched at the street level. Twenty-two subjects who listed a post office box address were excluded from the analysis. We used the Area Deprivation Index (ADI) to describe neighborhood disadvantage. Of the 17 indicators used in the original ADI, we removed two indicators: the percentage of households without a telephone, and the percentage of occupied housing units without complete plumbing. This was done as they showed little variation across Northeast Ohio. We then used the R “Sociome” package [41] to estimate the ADI for each census tract in Ohio. Sociome uses locally-derived factor score coefficients to weight the 15 census indicators that comprise the ADI. Because our study data spans the time period 2012 to 2014, we chose to use the 2015 5-year American Community Survey which includes the years 2011 to 2015 as our data source for calculating the ADI. The stability of the ADI over time is not well defined, however US census-based estimates are believed to be stable at the neighborhood level and the bivariate association between the 2009 and 2017 ADI for Ohio has an estimated correlation of 0.94, suggesting minimal variation over time [42].

Tests of differences (t-tests and χ^2^ tests for continuous and categorical outcomes, respectively) of demographic and clinical variables were calculated across the median ADI of the study population. Outcomes included postpartum sterilization achievement within 90 days of delivery, time in days to sterilization achievement from day of delivery, postpartum visit attendance within 90 days of delivery, and subsequent pregnancy within 365 days of delivery. Subsequent pregnancy was defined as either positive urine or serum pregnancy test, presentation for prenatal care, or notation in our hospital’s clinical documentation of pregnancy care at an outside hospital.

Differences in proportions of the three categorical outcomes across ADI were compared via χ^2^ with Yates’ continuity correction and odds ratio (OR) with 95% confidence intervals (CI). Differences in time to sterilization with 95% CIs across ADI were calculated using Cox proportional hazards regression. For these outcomes, the association with neighborhood disadvantage was analyzed across median ADI as well as in quartiles of ADI to better understand both the bivariate as well as spectrum effect, if any, of increasing limits on neighborhood resources and study outcomes.

Given relevant potential confounding between insurance type and ADI as well as the additional federal policy barrier that patients with Medicaid but not private insurance face, we also stratified our analyses of the association of neighborhood disadvantage and study outcomes by insurance type (Medicaid versus private). This was done by conducting univariable logistic regression of categorical outcomes and ADI and univariable Cox proportional hazards regression for time to sterilization for patients with Medicaid and patients with private insurance.

All tests were two-tailed and an alpha of 0.05 was used to define statistical significance. All analyses were performed using R Version 3.4.0. Records were abstracted and iteratively coded by four trained researchers (JM, BW, EV, MM). The Fleiss’ kappa score for concordance was 0.91 for the plan of sterilization between the four researchers. We were missing data in 20 (1.5%) records for the variable of adequacy of prenatal care, 34 (2.6%) records for marital status, and 56 (4.2%) records for education level. Complete data were available for 1228 (92.2%) records. In a post-hoc power calculation of this secondary analysis, using the sample sizes and proportions of patients in this study achieving postpartum sterilization, we calculated that we had a power of 0.89 for the association between sterilization achievement across median ADI. This study was approved by the institutional review board of the MetroHealth Medical Center.

## Results

One thousand three hundred thirty-two patients were included in this analysis. Clinical and demographic characteristics of those patients desiring sterilization stratified by the median ADI are presented in Table 1. Briefly, those patients living in areas above the median ADI (i.e., areas with higher disadvantage) were younger, more parous, more likely to have delivered vaginally, less likely to have adequate prenatal care, more likely to be Black, less likely to be married, less likely to have attended college, and more likely to have Medicaid insurance.

**Table 1:** Clinical and demographic characteristics of patients desiring postpartum sterilization by median of area deprivation index (ADI)

Variable	Neighborhood Disadvantage		*p*-value
	Above the Median ADI ≥ 126^a^ *n* = 667	Below the Median ADI < 126 *n* = 665	
Mean maternal age at delivery (years)	29.2 (5.4)	31.2 (5.3)	< 0.001
Parity ≥2	535 (80.2)	483 (72.6)	0.003
Median Gestational age at delivery (weeks)	39.0 (37.0–41.0)	39.0 (37.0–41.0)	0.94
Route of Delivery			< 0.001
Spontaneous vaginal delivery	409 (61.3)	332 (49.9)	
Operative vaginal delivery	15 (2.2)	13 (2.0)	
Cesarean section	243 (36.4)	320 (48.1)	
Adequate prenatal care (≥6 visits)	511 (76.6)	535 (80.5)	0.21
(Missing)	5 (1.3)	0 (0)	
Race and Ethnicity			< 0.001
Black or African American	396 (59.4)	271 (40.8)	
White	143 (21.4)	259 (38.9)	
Hispanic	112 (16.8)	100 (15.0)	
Asian	4 (0.6)	9 (1.4)	
Other	12 (1.8)	26 (3.9)	
Married	84 (12.6)	214 (32.2)	< 0.001
(Missing)	13 (3.3)	0 (0.0)	
Attended College	136 (20.4)	274 (41.2)	< 0.001
(Missing)	23 (5.8)	3 (4)	
Insurance			< 0.001
Private	24 (3.6)	131 (19.7)	
Medicaid	573 (85.9)	456 (68.6)	
Medicare	58 (8.7)	49 (7.4)	
None	18 (2.1)	23 (5.0)	

Presented as n (%), mean (SD), or median (IQR)

^a^ Higher ADI associated with greater neighborhood disadvantage

Clinical and demographic characteristics of the study population across quartiles of ADI are shown in Table 2. As ADI increased, patients were more likely to be younger, more parous, more likely to have delivered vaginally, less likely to have adequate prenatal care, more likely to be Black, less likely to be married, less likely to have attended college, and more likely to have Medicaid insurance. The relationship between sterilization achievement, time to sterilization postpartum visit attendance, subsequent pregnancy and ADI by quartile are also presented in Table 2. As ADI increased, patients were less likely to undergo their desired postpartum sterilization procedures, more likely to have a longer time to sterilization, less likely to attend their postpartum visit, and more likely to have a subsequent pregnancy.

**Table 2:** Clinical and demographic characteristics of patients desiring postpartum sterilization by quartile of area deprivation index (ADI)

Variable	Neighborhood Disadvantage			
	Quartile of ADI^a^			
	First (57–111) *n* = 332	Second (111–126) *n* = 333	Third (126–137) *n* = 370	Fourth (137–176) *n* = 297
Mean maternal age at delivery (years)	32.1 (5.0)	30.3 (5.3)	29.3 (5.5)	29.0 (5.3)
Parity ≥2	223 (67.2)	260 (78.1)	291 (78.6)	244 (82.2)
Mean Gestational age at delivery (weeks)	38.0 (2.2)	37.7 (2.6)	37.8 (2.6)	37.7 (2.8)
Route of delivery				
Spontaneous vaginal delivery	157 (47.3)	175 (52.6)	228 (61.6)	181 (60.9)
Operative vaginal delivery	4 (1.2)	9 (2.7)	8 (2.2)	7 (2.4)
Cesarean section	171 (51.5)	149 (44.7)	134 (36.2)	109 (36.7)
Adequate prenatal care (≥6 visits)	273 (82.2)	262 (78.7)	290 (78.4)	221 (74.4)
Race and ethnicity				
Black or African American	91 (27.4)	180 (54.1)	168 (45.4)	228 (76.8)
White	179 (53.9)	80 (24.0)	111 (30.0)	32 (10.8)
Hispanic	37 (11.1)	63 (18.9)	84 (22.7)	28 (9.4)
Asian	7 (2.1)	2 (0.6)	1 (0.3)	3 (1.0)
Other	18 (5.4)	8 (2.4)	6 (1.6)	6 (2.0)
Married	150 (45.2)	64 (19.2)	54 (14.6)	30 (10.1)
Attended college	169 (50.9)	105 (31.5)	82 (22.2)	54 (18.2)
Insurance				
Private	101 (30.4)	30 (9.0)	16 (4.3)	8 (2.7)
Medicaid	189 (56.9)	267 (80.2)	311 (84.1)	262 (88.2)
Medicare	24 (7.2)	25 (7.5)	33 (8.9)	25 (8.4)
None	18 (5.4)	11 (3.3)	10 (2.7)	2 (0.7)
Received postpartum sterilization	194 (58.4)	162 (48.6)	172 (46.5)	127 (42.8)
Postpartum visit attendance	246 (74.1)	212 (63.7)	226 (61.1)	167 (56.2)
Subsequent pregnancy	22 (6.6)	47 (14.1)	47 (12.7)	54 (18.2)

Presented as n (%), mean (SD), or median (IQR)

^a^ Higher ADI associated with greater neighborhood disadvantage

Association of ADI and study outcomes are shown in Table 3. Briefly, patients living in areas with above the median ADI were less likely to obtain postpartum sterilization within 90 days of delivery (OR 0.84, 95% CI 0.75–0.93). Median time to sterilization was longer for those living in areas with ADI above the median (HR 0.81, 95% CI 0.70–0.95). Patients living in census tracts with ADI at or above the median were less likely to attend their postpartum visit (OR 0.86, CI 0.79–0.93). Finally, patients living in areas with ADI at or above the median were more likely to have a subsequent pregnancy within a year of the index delivery (OR 1.46, 95% CI 1.10–1.94).

**Table 3:** Association of Neighborhood Disadvantage with Sterilization Outcomes

Outcome	Neighborhood Disadvantage		OR (95% CI)
	Above the Median ADI ≥ 126^a^ n = 667	Below the Median ADI < 126 n = 665	
Obtained postpartum sterilization	299 (44.8)	356 (53.5)	0.84 (0.75–0.93)
Median time to sterilization (days)	77	56	0.81 (0.70–0.95)^a^
Postpartum visit attendance	393 (58.9)	458 (68.9)	0.86 (0.79–0.93)
Subsequent pregnancy within 365 days	101 (51.1)	69 (10.4)	1.46 (1.10–1.94)

Presented as n (%)

^a^ HR (95% CI)

In an insurance-stratified analysis in which ADI was analyzed continuously, greater ADI was significantly associated with lower sterilization achievement among patients with Medicaid, but not private insurance, (OR 0.92, 95% CI 0.86–0.98 vs OR 0.94, 95% CI 0.81–1.09). For both Medicaid and privately insured patients, there was no significant association between ADI and time to sterilization (Medicaid: HR 0.95, 95% CI 0.91–1.00; Private insurance: HR 0.97, 95% CI 0.89–1.07) or postpartum visit attendance (Medicaid: OR 0.94, 95% CI 0.88–1.00); Private insurance: OR 0.84, 95% CI 0.65–1.06). However, ADI was associated with subsequent pregnancy both for patients with Medicaid (OR 1.14, 95% CI 1.04–1.26) and those with private insurance (OR 1.89, 95% CI 1.28–3.15).

## Discussion

In our study of neighborhood disadvantage and postpartum sterilization, living in an area with higher neighborhood disadvantage was associated with a decreased rate of completion of desired postpartum sterilization, increased time to completion, decreased rate of postpartum visit attendance, and increased rate of subsequent short-interval pregnancy. To our knowledge, this study is the first to report association between neighborhood disadvantage and postpartum outcomes.

Non-completion of desired postpartum sterilization is detrimental to both individual and public health in terms of patients reporting feeling frustrated, angry, and anxious as well as their increased risk of subsequent short-interval pregnancy [9]. Such short-interval pregnancy carries a higher risk of preterm birth, which has also been shown to be associated with neighborhood disadvantage [35, 36, 38]. Racial/ethnic disparities in neighborhood disadvantage exist in our study and have been previously demonstrated in perinatal health outcomes, including maternal mortality [43–46]. Therefore, addressing social determinants of health may play a powerful role in reducing non-fulfillment of desired postpartum sterilization procedures, overall perinatal morbidity and mortality, as well as racial/ethnic inequities in health outcomes due to unfilled sterilization requests.

Increasing neighborhood disadvantage was associated with an increased likelihood of having Medicaid insurance. Patients with Medicaid, but not those with private insurance, face additional policy-level barriers to receipt of desired postpartum sterilization [4]. As demonstrated in our stratified analysis, for both patients with Medicaid and private insurance, living in areas of higher neighborhood disadvantage is associated with decreased likelihood of postpartum sterilization completion, although this was statistically significant only for patients with Medicaid. Thus, it is possible that those patients with Medicaid living in areas of higher neighborhood disadvantage were less likely to overcome the policy barrier of the required waiting period. However, other potential differences in the two populations as well as differences in health delivery could also contribute to this difference and should be further studied.

The major strength of this study is that it is a planned secondary analysis from a detailed and large data set including all deliveries over a 3-year period. However, the study has several limitations. The study is retrospective and is therefore limited by the quality of the medical records. For example, we cannot account for those patients who had subsequent pregnancies within the study period but obtained prenatal care or termination of pregnancy outside our institution. As a single institution study, our patient demographics, physician practices, and institutional policies surrounding sterilization may impact generalizability. We operationalized neighborhood disadvantage using the ADI, a composite index based on census-area indicators. Therefore, limitations of this analysis include potential confounding of study demographics with variables included in the index as well as the fact that we used a revised version of the ADI given our study population. Thus, further work using larger samples and additional patient- and family-level data will be necessary to better understand and disentangle individual and neighborhood level effects [47, 48]. Finally, several associations identified in our models are within the zone of potential bias and therefore should be interpreted with caution [49].

## Conclusion

In conclusion, we found that neighborhood disadvantage is inversely associated with the rate of desired postpartum sterilization, especially for patients with Medicaid insurance. Despite the limitations of the area deprivation index, our study demonstrates the association of social determinants of health with sterilization outcomes. Thus, while revising the Medicaid sterilization policy is important to reduce barriers to care, further study into the association of social determinants of health in reproductive health is also warranted given the impact on both individual patient and public health outcomes. Improving public health systems through addressing structural inequities such as decreased distance to clinic and improved hours of access as well as assistance with travel and childcare coordination is necessary to reduce financial and structural barriers to care.

## Data Availability

The datasets used and or/analyzed during the current study are not publicly available due to patient privacy concerns but are available from the corresponding author on reasonable request after IRB permission is obtained.

## Abbreviations

ADI: Area Deprivation Index
OR: Odds Ratio
aOR: Adjusted Odds Ratio
CI: Confidence Interval
HR: Hazard Ratio

## References

[1] Daniels K, Daugherty J, Jones J, Mosher W (2015). Current Contraceptive Use and Variation by Selected Characteristics Among Women Aged 15–44: United States, 2011–2013.

[2] American College of Obstetricians and Gynecologists. Committee opinion no. 530: Access to postpartum sterilization. Obstet Gynecol 2012;120(1):212–215. https://www.acog.org/-/media/Committee-Opinions/Committee-on-Health-Care-for-Underserved-Women/co530.pdf?dmc=1&ts=20190312T1206586056. Accessed March 12, 2019. 10.1097/AOG.0b013e318262e354 22914423

[3] Zite N, Wuellner S, Gilliam M (2005). Failure to obtain desired postpartum sterilization: risk and predictors. Obstet Gynecol.

[4] Borrero S, Zite N, Potter JE, Trussell J (2014). Medicaid policy on sterilization — anachronistic or still relevant?. N Engl J Med.

[5] Committee on Ethics (2017). Committee Opinion No. 695: sterilization of women: ethical issues and considerations. Obstet Gynecol.

[6] Brown BP, Chor J (2014). Adding injury to injury: ethical implications of the medicaid sterilization consent regulations. Obstet Gynecol.

[7] Moaddab A, Mccullough LB, Chervenak FA (2015). Health care justice and its implications for current policy of a mandatory waiting period for elective tubal sterilization. Am J Obstet Gynecol.

[8] Petchesky RP (1979). Reproduction, ethics, and public policy: the Federal Sterilization Regulations. Hast Cent Rep.

[9] Block-Abraham D, Arora KS, Tate D, Gee RE (2015). Medicaid consent to sterilization forms. Clin Obstet Gynecol.

[10] Arora KS, Wilkinson B, Verbus E (2018). Medicaid and fulfillment of desired postpartum sterilization. Contraception..

[11] Heaman MI, Sword W, Elliott L, et al. Barriers and facilitators related to use of prenatal care by inner-city women: perceptions of health care providers. BMC Pregnancy Childbirth. 2015;15(1). 10.1186/s12884-015-0431-5. 10.1186/s12884-015-0431-5 PMC4302607 25591945

[12] Heaman MI, Moffatt M, Elliott L, et al. Barriers, motivators and facilitators related to prenatal care utilization among inner-city women in Winnipeg, Canada: a case–control study. BMC Pregnancy Childbirth. 2014;14(1). 10.1186/1471-2393-14-227. 10.1186/1471-2393-14-227 PMC4223395 25023478

[13] Messer LC, Laraia BA, Kaufman JS (2006). The development of a standardized neighborhood deprivation index. J Urban Health.

[14] Singh GK (2003). Area deprivation and widening inequalities in US mortality, 1969-1998. Am J Public Health.

[15] Bachmann JM, Huang S, Gupta DK (2017). Association of Neighborhood Socioeconomic Context with Participation in cardiac rehabilitation. J Am Heart Assoc.

[16] Al Adas Z, Nypaver TJ, Shepard AD (2019). Survival after abdominal aortic aneurysm repair is affected by socioeconomic status. J Vasc Surg.

[17] Tod E, McCartney G, Fischbacher C (2019). What causes the burden of stroke in Scotland? A comparative risk assessment approach linking the Scottish vHealth Survey to administrative health data. PLOS ONE.

[18] Honjo K, Iso H, Nakaya T (2015). Impact of neighborhood socioeconomic conditions on the risk of stroke in Japan. J Epidemiol.

[19] Marcus JL, Hurley LB, Chamberland S (2018). Disparities in Initiation of Direct-Acting Antiviral Agents for Hepatitis C Virus Infection in an Insured Population. Public Health Rep.

[20] Putrik P, Ramiro S, Orueta JF (2018). Socio-economic inequalities in occurrence and health care costs in rheumatic and musculoskeletal diseases: results from a Spanish population-based study including 1.9 million persons. Clin Exp Rheumatol.

[21] Sapra KJ, Yang W, Walczak NB, Cha SS. Identifying High-Cost Medicare Beneficiaries: Impact of Neighborhood Socioeconomic Disadvantage. Popul Health Manag. 2019. doi: 10.1089/pop.2019.0016. 10.1089/pop.2019.0016 31207198

[22] Hu J, Kind AJH, Nerenz D (2018). Area deprivation index predicts readmission risk at an urban teaching hospital. American journal of medical quality : the official journal of the American College of Medical Quality.

[23] Vesoulis ZA, Lust CE, Cohlan BA, Liao SM, Mathur AM. Poverty and Excess Length of Hospital Stay in Neonatal Opioid Withdrawal Syndrome. J Addict Med. 2019. 10.1097/ADM.0000000000000540. 10.1097/ADM.0000000000000540 PMC6881526 31149915

[24] Warren Andersen S, Blot WJ, Shu X-O (2018). Associations between neighborhood environment, health behaviors, and mortality. Am J Prev Med.

[25] Li X, Sundquist J, Zöller B, Sundquist K (2015). Neighborhood deprivation and lung cancer incidence and mortality: a multilevel analysis from Sweden. J Thorac Oncol.

[26] Chan T-C, Chiang P-H, Su M-D, Wang H-W, Liu MS (2014). Geographic disparity in chronic obstructive pulmonary disease (COPD) mortality rates among the Taiwan population. PLoS One.

[27] Padilla CM, Deguen S, Lalloue B (2013). Cluster analysis of social and environment inequalities of infant mortality. A spatial study in small areas revealed by local disease mapping in France. Sci Total Environ.

[28] Tobias M, Jackson G (2001). Avoidable mortality in New Zealand, 1981-97. Aust N Z J Public Health.

[29] Dolk H, Thakrar B, Walls P (1999). Mortality among residents near cokeworks in Great Britain. Occup Environ Med.

[30] van Jaarsveld CHM, Miles A, Wardle J (2007). Pathways from deprivation to health differed between individual and neighborhood-based indices. J Clin Epidemiol.

[31] Lantos PM, Hoffman K, Permar SR (2018). Neighborhood disadvantage is associated with high Cytomegalovirus Seroprevalence in pregnancy. J Racial Ethn Health Disparities.

[32] Deguen S, Kihal W, Jeanjean M, Padilla C, Zmirou-Navier D (2016). Neighborhood Deprivation and Risk of Congenital Heart Defects, Neural Tube Defects and Orofacial Clefts: A Systematic Review and Meta-Analysis. PLOS ONE.

[33] Wentz AE, Messer LC, Nguyen T, Boone-Heinonen J (2014). Small and large size for gestational age and neighborhood deprivation measured within increasing proximity to homes. Health Place.

[34] Deguen S, Ahlers N, Gilles M (2018). Using a clustering approach to investigate socio-environmental inequality in preterm birth—a study conducted at fine spatial scale in Paris (France). Int J Environ Res Public Health.

[35] Kramer MR, Dunlop AL, Hogue CJR (2014). Measuring women’s cumulative neighborhood deprivation exposure using longitudinally linked vital records: a method for life course MCH research. Matern Child Health J.

[36] Ma X, Fleischer NL, Liu J, Hardin JW, Zhao G, Liese AD (2015). Neighborhood deprivation and preterm birth: an application of propensity score matching. Ann Epidemiol.

[37] Schempf AH, Kaufman JS, Messer LC, Mendola P (2011). The neighborhood contribution to black-white perinatal disparities: an example from two North Carolina counties, 1999-2001. Am J Epidemiol.

[38] O’Campo P, Burke JG, Culhane J (2008). Neighborhood deprivation and preterm birth among non-Hispanic black and white women in eight geographic areas in the United States. Am J Epidemiol.

[39] Morris J, Ascha M, Wilkinson B, Verbus E, Montague M, Mercer BM (2019). Desired sterilization procedure at the time of cesarean delivery according to insurance status. Obstet Gynecol.

[40] Santos NET, Oliveira AE, Zandonade E, Leal MC (2013). Access to prenatal care: assessment of the adequacy of different indices. Cad Saude Publica.

[41] Krieger N, Dalton J, Wang C, Perzynski A. Sociome: Operationalizing Social Determinants of Health Data for Researchers. Version: 0.3.4. https://cran.rproject.org/ web/packages/sociome/index.html. Accessed May 11, 2020.

[42] Adie Y, Kats DJ, Tlimat A, Perzynski A, Dalton J, Gunzler D (2020). Neighborhood disadvantage and lung cancer incidence in ever-smokers at a safety net health-care system: a retrospective study. Chest..

[43] Thiel de Bocanegra H, Braughton M, Bradsberry M, Howell M, Logan J, Schwarz EB (2017). Racial and ethnic disparities in postpartum care and contraception in California’s Medicaid program. Am J Obstet Gynecol.

[44] Dehlendorf C, Park SY, Emeremni CA, Comer D, Vincett K, Borrero S (2014). Racial/ethnic disparities in contraceptive use: variation by age and women’s reproductive experiences. Am J Obstet Gynecol.

[45] Schummers L, Hacker MR, Williams PL, Hutcheon JA, Vanderweele TJ, McElrath TF, Hernandez-Diaz S (2019). Variation in relationships between maternal age at first birth and pregnancy outcomes by maternal race: a population-based cohort study in the United States. BMJ Open.

[46] Mehta PK, Kieltyka L, Bacchuber MA, Smiles D, Wallace M, Zapata A, Gee RE. Racial inequities in preventable pregnancy-related deaths in Louisiana, 2011–2016. Obstet Gynecol. 2020; Epub ahead of print. 10.1097/AOG.0000000000003591 PMC7299502 31923055

[47] Geronimus AT, Bound J, Neidert LJ (1996). On the validity of using census geocode characteristics to proxy individual socioeconomic characteristics. J AM Statist Assoc.

[48] Geronimus AT, Bound J (1998). Use of census based aggregate variables to proxy for socio-economic group: evidence from national samples. Am J Epidemiol.

[49] Grimes DA, Schulz KF (2012). False alarms and pseudo-epidemics. Obstet Gynecol.

